# Prevalence and determinants of asthenopia among ophthalmologists in China: a national cross-sectional survey

**DOI:** 10.3389/fpubh.2023.1290811

**Published:** 2023-12-29

**Authors:** Na Lin, Yu Zhu, Xiaotian Wu, Maoyuan Yang, Fan Lu, Ruzhi Deng

**Affiliations:** ^1^National Clinical Research Center for Ocular Diseases, Eye Hospital, Wenzhou Medical University, Wenzhou, China; ^2^School of Ophthalmology and Optometry, Wenzhou Medical University, Wenzhou, China

**Keywords:** asthenopia, ophthalmologists, occupational health, prevalence, risk factors

## Abstract

**Introduction:**

The role of ophthalmologists is defined by tasks requiring visual effort, emphasizing the importance of examining their condition within the realm of occupational visual health. Our goal was to explore the occurrence of asthenopia among Chinese ophthalmologists and identify contributing factors through the use of a reliable and validated survey instrument.

**Methods:**

A national cross-sectional online survey was carried out in June 2017, involving 6,220 practicing ophthalmologists in China. Utilizing an 11-item Asthenopia Survey Questionnaire with established reliability and validity. Prevalence rates of asthenopia among subgroups categorized by age, gender, hospital classification, physician level, daily near vision activity duration, sleep duration, sleep quality, presbyopia status, and history of eye surgery were determined using the independent *t*-test, chi-square test and bonferroni test. Multiple logistic regression analysis was employed to pinpoint independent factors linked to asthenopia.

**Results:**

Out of the 5,009 ophthalmologists who completed the survey, a 40.7% prevalence of asthenopia was identified. Multivariate analysis revealed that good sleep quality (OR: 0.24, 95%CI: 0.20–0.30), moderate sleep quality (OR: 0.47, 95%CI: 0.38–0.59), engaging in daily near vision activities for less than 7 h (OR: 0.76, 95%CI: 0.68–0.86), having daily sleep duration exceeding 7 h (OR: 0.87, 95%CI: 0.77–0.98), and working in tertiary hospitals (OR: 0.88, 95%CI: 0.78–0.99) were protective factors against asthenopia. Conversely, presbyopia was identified as a risk factor (OR: 1.33, 95%CI: 1.04–1.70). All calculated *p* values were below 0.05. Age, gender, physician level, and eye surgery history were not related factors.

**Conclusion:**

Asthenopia is prevalent among Chinese ophthalmologists, with employment in tertiary hospitals providing a protective effect and presbyopia is a risk factor. Preventive strategies include improving sleep quality, restricting daily near vision activity to under 7 h, and extending daily sleep duration to over 7 h. Further investigation is needed to explore the protective implications of working in tertiary hospitals.

## 1 Introduction

Asthenopia, characterized by visual fatigue or eye strain, is a prevalent vision disorder affecting a substantial portion of the population (1). This syndrome involves subjective sensations of ocular, visual, or systemic discomfort, significantly impacting attention, work capacity, and overall performance (2, 3). The widespread integration of digital devices into daily life has increased the risk of asthenopia for individuals across all age groups (4–7).

A notable shift in the contemporary workforce is the increased visual demands, especially concerning near-range vision in diverse work environments (8). Within occupational settings, asthenopia emerges as a prominent ocular condition (9, 10). Ophthalmologists, distinct in their visual occupational health challenges compared to other medical professionals, undertake intricate tasks requiring substantial visual effort. Their activities include operating in dimly lit conditions, utilizing computers, slit lamps, and surgical microscopes. Their specialized knowledge uniquely positions them to prevent, alleviate, and promptly address asthenopia.

While previous researches have explored asthenopia prevalence, limited attention has been given to its occurrence among medical professionals and undergraduate students. A study in Lisbon involving 27 ophthalmologists reported a prevalence of 92.6% (11). Similarly, a study in Kathmandu with 208 undergraduate medical students during the COVID-19 pandemic revealed a prevalence of 90.8% (12). Earlier investigations have linked asthenopia to personal and environmental factors, such as advancing age (4), prolonged periods of near vision tasks (7), emotional states (13), and lighting mismatches between the screen and surroundings (14). The identification of risk and protective factors related to asthenopia is crucial. While previous studies primarily relied on questionnaires, allowing individuals to more accurately assess their symptoms, no study to date has explored asthenopia prevalence and risk factors among Chinese ophthalmologists.

With this context, we conducted a national survey to investigate the prevalence and determinants of asthenopia among ophthalmologists in China, utilizing a reliable and validated survey tool.

## 2 Materials and methods

### 2.1 Study design

This cross-sectional nationwide web-based survey was conducted in June 2017. Invitations were distributed through the DoctorCircle physician community software (Shenzhen, China) to 6,220 registered ophthalmologists practicing in China. Anonymous data collection was facilitated using the online survey tool WJX (Changsha, China), and the survey remained open for 1 week.

### 2.2 Ethical approval

Ethical clearance for this study was obtained from the Institutional Ethics Committee of The Eye Hospital of Wenzhou Medical University (Approval No. KYK-2016-8). The study adhered to the principles of the Declaration of Helsinki. Participants received information regarding the survey’s purpose, significance, content, and privacy protection before engaging in the survey.

### 2.3 Sample size calculation

The required minimum sample size was calculated using the formula: $n=\frac{z^{2}p\left(1-p\right)}{E^{2}}$ , where *Z* = 1.96 (95% confidence interval), P was set at 12.1% based on a similar survey in China (6), and *E* = 0.1 × P. Thus, the minimum sample size was determined to be 2,791.

### 2.4 The questionnaire

**Figure 1:**
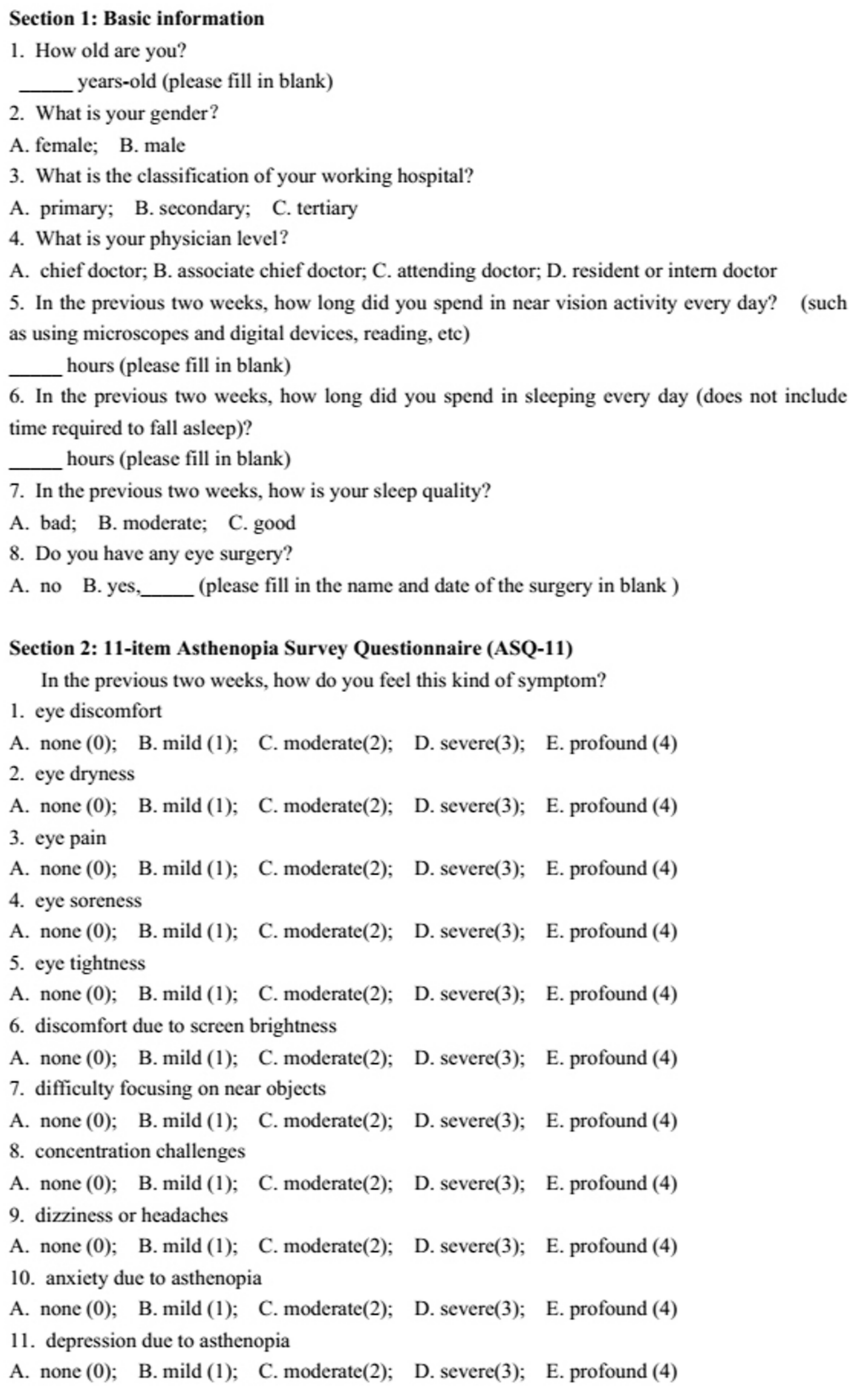
The survey questionnaire form.

The questionnaire, illustrated in Figure 1, comprised two sections. The initial section collected demographic and work pattern information, encompassing age, gender, institution type, and physician level. Participants provided details on their average duration of near vision activities in the past 2 weeks, involving the use of microscopes and digital devices, along with information on sleep duration, quality, and any history of eye surgery.

The second section utilized the 11-item Asthenopia Survey Questionnaire (ASQ-11), previously developed by our research group has been acknowledged as a reliable and validated survey instrument for assessing asthenopia among the Chinese population (15, 16), to evaluate asthenopia. Rooted in classical test theory, the ASQ-11 was crafted based on a literature review and underwent validation through expert consensus, pre-testing, pilot testing, and re-evaluation. Participants assessed the occurrence of 11 ocular, visual, and systemic symptoms over the previous 2 weeks. These symptoms included Q1: eye discomfort, Q2: eye dryness, Q3: eye pain, Q4: eye soreness, Q5: eye tightness, Q6: discomfort due to screen brightness, Q7: difficulty focusing on near objects, Q8: concentration challenges, Q9: dizziness or headaches, Q10: anxiety due to asthenopia, and Q11: depression due to asthenopia.

Each question employed a five-point response scale, ranging from “none” (0), “mild” (1), “moderate” (2), “severe” (3), to “profound” (4). The total score of the questionnaire equated to the sum of individual question scores. Participants with a total score of 9 or higher were categorized as experiencing asthenopia, with a sensitivity of 74.19% and a specificity of 80.65% as detailed in our previous publication (16).

The survey was administered online, permitting a single IP address to respond once. Response time was restricted to 1–10 min based on the previous reported average completion time (2.82 ± 0.43) mins, with submissions beyond this range considered invalid.

### 2.5 Statistical analysis

Statistical analyses were conducted using SPSS 25.0 software (SPSS Inc., Chicago, IL). Continuous variables were presented as mean ± standard deviation or as median (interquartile range, IQR). Categorical variables were expressed as counts and percentages. The durations of near vision activity and sleep were categorized into two groups (≤7 h per day and > 7 h per day) based on their median values. Their presbyopia status was categorized as yes (≥45-years-old) and no (<45-years-old) based on the age when negative patient impact of presbyopia on visual function and quality of life in previous reports (17, 18). Associations between different factors and asthenopia prevalence were assessed using the independent *t*-test or chi-square test or bonferroni test. Factors with *p* values below 0.1 were included in the forward likelihood ratio method of multiple logistic regression analysis. A value of *p* less than 0.05 was considered statistically significant.

## 3 Results

In this study, 6,220 questionnaires were distributed, with 5,578 (89.7%) being successfully retrieved. Among these, 5,009 (80.5%) questionnaires from 28 regions across China were considered valid, while 569 were deemed invalid due to demographic information omissions (232 cases), missing asthenopia-related symptom responses (253 cases), and response times exceeding the designated range (84 cases). Examination of the 232 demographic information incomplete questionnaires revealed no substantial divergence in asthenopia prevalence compared to the calculated rate from the valid 5,009 questionnaires (40.1% vs. 40.7%, *p* = 0.031).

### 3.1 Asthenopia and demographic factors

The overall prevalence of asthenopia among the 5,009 participating ophthalmologists was 40.7%. Participant ages ranged from 22 to 60 years, with a mean age of 35.2 ± 8.6 years. As detailed in Table 1, the majority were females (60.8%), approximately half (49.9%) worked in tertiary hospitals, and 37.4% were attending doctors. Median durations of near vision activity and sleep were 7 h per day (IQR: 5.0; 9.0) and 7 h per day (IQR: 7.0; 8.0), respectively. Sleep quality was reported as moderate by 43.0% of participants and good by 48.2%. 18.1% participants were presbyopia and only 5.7% reported a history of eye surgery.

**Table 1:** Participant demographics.

Variables	Total (*n* = 5,009)	Asthenopia (*n* = 2,039)	Non-asthenopia (*n* = 2,970)	t orχ^2^ value*	*p* value
Prevalence, %		40.7	59.3		
Age, years (range)	35.2 ± 8.6 (22–60)	35.5 ± 8.9 (22–60)	35.0 ± 8.4 (22–60)	2.105^a^	**0.035**
Gender, *n*(%)				0.048^b^	0.827
Female	3,043 (60.8)	1,235 (60.6)	1,808 (60.9)		
Male	1,966 (39.2)	804 (39.4)	1,162 (39.1)		
Classification of hospital, *n*(%)				4.114^b^	**0.043**
Non-tertiary	2,510 (50.1)	1,057 (51.8)	1,453 (48.9)		
Tertiary	2,499 (49.9)	982 (48.2)	1,517 (51.1)		
Duration of near vision activity, *n*(%)				31.425^b^	**<0.001**
>7 h per day	2,321 (46.3)	1,042 (51.1)	1,279 (43.1)		
≤7 h per day	2,688 (53.7)	997 (48.9)	1,691 (56.9)		
Physician level, *n*(%)				15.057^b,c^	**0.002**
Chief	312 (6.2)	153 (7.5)	159 (5.4)		
Associate chief	957 (19.1)	408 (20.0)	549 (18.5)		
Attending	1,871 (37.4)	716 (35.1)	1,155 (38.9)		
Resident or intern	1,869 (37.3)	762 (37.4)	1,107 (37.2)		
Sleep duration, *n*(%)				27.244^b^	**<0.001**
≤7 h per day	3,125 (62.4)	1,360 (66.7)	1,765 (59.4)		
>7 h per day	1,884 (37.6)	679 (33.3)	1,205 (40.6)		
Sleep quality, *n*(%)				248.206^b,d^	**<0.001**
Bad	439 (8.8)	289 (14.2)	150 (5.1)		
Moderate	2,156 (43.0)	1,009 (49.5)	1,147 (38.6)		
Good	2,414 (48.2)	741 (36.3)	1,673 (56.3)		
Presbyopia status, *n*(%)				8.644^b^	**0.003**
No	4,101 (81.9)	1,630 (79.9)	2,471 (83.2)		
Yes	908 (18.1)	409 (20.1)	499 (16.8)		
History of eye surgery, *n*(%)				0.005^b^	0.943
No	4,723 (94.3)	1,922 (94.3)	2,801 (94.3)		
Yes	286 (5.7)	117 (5.7)	169 (5.7)		

*Comparison of demographic information of participants with asthenopia and non-asthenopia.

^a^ Independent *t*-test; ^b^ Chi-square test; ^c^ Bonferroni test showed the prevalence of asthenopia among chief doctors was significantly higher than other level doctors (all *p* values < 0.05), and there were no statistical differences between every two subgroups of other level doctors (all *p* values > 0.05). Hence, the physician level was categorized as “chief doctors” and “associate chief doctors and below” in the next multivariate analysis; ^d^ Bonferroni test showed significant differences of the prevalence of asthenopia between every two subgroups of sleep quality (all *p* values < 0.05).

t or χ^2^ value with statistically significant were listed in bold.

Furthermore, asthenopia prevalence was significantly lower among younger ophthalmologists, those working in tertiary hospitals and chief doctors, those engaging in less than 7 h of daily near vision activity, individuals sleeping more than 7 h per day, with good or moderate sleep quality, and without presbyopia. No significant prevalence difference was observed based on gender or eye surgery history, as indicated in Table 1.

### 3.2 Multivariate analysis of selected factors

After multivariate analysis (all *p* values <0.05), six factors emerged significantly associated with asthenopia, as presented in Table 2. The most potent protective factor was good sleep quality (OR: 0.24, 95% CI: 0.20–0.30), followed by moderate sleep quality (OR: 0.47, 95% CI: 0.38–0.59), engaging in less than 7 h of daily near vision activity (OR: 0.76, 95% CI: 0.68–0.86), sleeping over 7 h per day (OR: 0.87, 95% CI: 0.77–0.98), and working in tertiary hospitals (OR: 0.88, 95% CI: 0.78–0.99). Presbyopia (OR: 1.33, 95% CI: 1.04–1.70) was identified as a risk factor. Advancing age (OR: 0.99, 95% CI: 0.98–1.01, *p* = 0.344) and physician level (OR: 0.75, 95% CI: 0.56–1.06, *p* = 0.065) were not related after multivariate adjusting.

**Table 2:** Factors associated with asthenopia – multivariate binary logistic regression analysis.

Variables	OR (95% CI)	*p* value
Age, years	0.99 (0.98–1.01)	0.344
Classification of hospital		
Non-tertiary	Reference	
Tertiary	0.88 (0.78–0.99)	**0.027**
Physician level		
Chief	Reference	
Associate chief and below	0.75 (0.56–1.06)	0.065
Duration of near vision activity		
>7 h per day	Reference	
≤7 h per day	0.76 (0.68–0.86)	**<0.001**
Sleep duration		
≤7 h per day	Reference	
>7 h per day	0.87 (0.77–0.98)	**0.022**
Sleep quality		
Bad	Reference	
Moderate	0.47 (0.38–0.59)	**<0.001**
Good	0.24 (0.20–0.30)	**<0.001**
Presbyopia status		
No	Reference	
Yes	1.33 (1.04–1.70)	**0.025**

OR, odd ratio; CI, confidence interval.

OR with statistically significant were listed in bold.

### 3.3 Comparison of related factors between ophthalmologists working in tertiary and non-tertiary hospitals

The prevalence of asthenopia was lower among ophthalmologists working in tertiary hospitals compared to non-tertiary hospitals (39.3% vs. 42.1%, *p* = 0.043). As outlined in Table 3, ophthalmologists working in tertiary hospitals were significantly younger (34.9 ± 8.6 years vs. 35.6 ± 8.5 years, *p* = 0.008), with a higher proportion engaging in over 7 h of daily near vision activity (47.8% vs. 44.9%, *p* = 0.036), and a lower proportion sleeping over 7 h per day (35.5% vs. 39.7%, *p* = 0.002). No statistical differences were observed in physician level, sleep quality, and presbyopia status between the two groups (*p* values were 0.329, 0.780 and 0.078, respectively).

**Table 3:** Comparison of factors between ophthalmologists in tertiary and non-tertiary hospitals.

Variables	Tertiary (n = 2,499)	Non-tertiary (*n =* 2,510)	t orχ^2^ value	*p* value
Age, years	34.9 ± 8.6	35.6 ± 8.5	−2.665^a^	**0.008**
Physician level, *n*(%)			0.952^a^	0.329
Chief	164 (6.6)	148 (5.9)		
Associate chief and below	2,335 (93.4)	2,365 (94.1)		
Duration of near vision activity, *n*(%)			4.408^b^	**0.036**
>7 h per day	1,195 (47.8)	1,126 (44.9)		
≤7 h per day	1,304 (52.2)	1,384 (55.1)		
Sleep duration, *n*(%)			9.178^b^	**0.002**
≤7 h per day	1,161 (64.5)	1,514 (60.3)		
>7 h per day	888 (35.5)	996 (39.7)		
Sleep quality, *n*(%)			0.496^b^	0.780
Bad	212 (8.5)	227 (9.0)		
Moderate	1,080 (43.2)	1,076 (42.9)		
Good	1,207 (48.3)	1,207 (48.1)		
Presbyopia status, *n*(%)			3.100^b^	0.078
No	2,070 (82.8)	2031 (80.9)		
Yes	429 (17.2)	479 (19.1)		

^a^ Independent *t*-test; ^b^ Chi-square test.

t or χ^2^ value with statistically significant were listed in bold.

### 3.4 Comparison of asthenopia symptoms between ophthalmologists working in tertiary and non-tertiary hospitals

**Figure 2:**
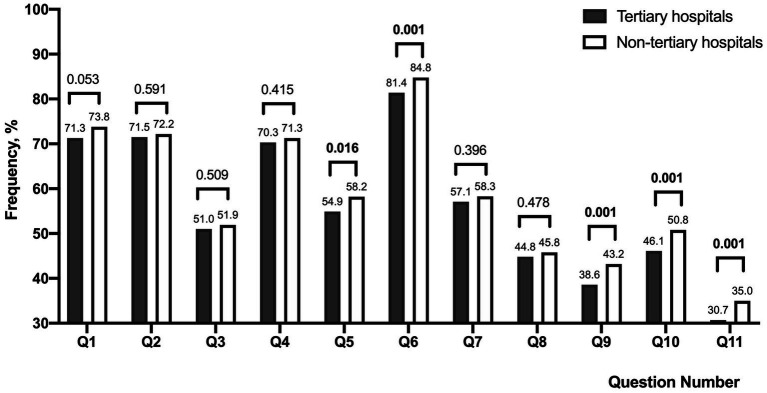
Illustration of the comparison of perceived asthenopia symptom frequencies among ophthalmologists in tertiary hospitals and non-tertiary hospitals (*n* = 5,009). The symptoms evaluated include Q1: eye discomfort, Q2: eye dryness, Q3: eye pain, Q4: eye soreness, Q5: eye tightness, Q6: discomfort due to screen brightness, Q7: difficulty focusing on near objects, Q8: concentration challenges, Q9: dizziness or headaches, Q10: anxiety due to asthenopia, and Q11: depression due to asthenopia. Chi-square testing revealed significantly lower frequencies of Q5, Q6, Q9, Q10, and Q11 among ophthalmologists in tertiary hospitals compared to those in non-tertiary hospitals (all *p* values < 0.05).

The occurrence of the following five asthenopia symptoms was lower among ophthalmologists in tertiary hospitals compared to non-tertiary hospitals (all *p* values <0.05): eye tightness (54.9% vs. 58.2%), discomfort due to screen brightness (81.4% vs. 84.8%), dizziness or headaches (38.6% vs. 43.2%), anxiety due to asthenopia (46.1% vs. 50.8%), and depression due to asthenopia (30.7% vs. 35.0%). Figure 2 illustrates the frequency of each asthenopia symptom perceived by ophthalmologists in the two groups.

## 4 Discussion

Ophthalmologists face unique occupational health challenges, particularly concerning asthenopia. Our study represents a groundbreaking application of the reliable and validated ASQ-11 survey tool among Chinese ophthalmologists, revealing a 40.7% prevalence of asthenopia within this professional group. According to the latest data from the China National Blindness Prevention Technical Steering Group, there are approximately 44,800 ophthalmologists nationwide (19), making our analysis encompass 11.2% (5,009) of this population. Significantly, our research identifies working in tertiary hospitals as a protective factor against asthenopia for Chinese ophthalmologists. Additionally, we recognize superior sleep quality, engaging in less than 7 h of daily near vision activity, sleeping more than 7 h per day as protective factors, while presbyopia emerges as a risk factor.

The prevalence of asthenopia in our group of ophthalmologist (40.7%) is notably lower than that reported among ophthalmologists in a university hospital in Lisbon (92.6%) (11). This difference can be attributed to variations in measurement tools; Lisbon’s study relied solely on self-reported eye strain and was limited to 27 participants, complicating the interpretation of its findings. Our large sample size enhances the reliability of our asthenopia prevalence estimation. Additionally, our observed prevalence among Chinese ophthalmologists is lower compared to other occupational groups: computer operators (46.3%) (20), Chinese college students (53.3%) (5), myopic individuals (57.0%) (21), radiologists (65.4%) (3), university instructors (70.4%) (22), bank employees (74.6%) (23), and information technology professionals (82.5%) (24). The lower prevalence among Chinese ophthalmologists may be attributed to a more precise and strict definition of asthenopia in this study, or their comprehensive knowledge of ophthalmology, likely contributing to effective asthenopia prevention. It is important to note that comparing asthenopia prevalence across different professions or populations is challenging. Standardized scoring systems and potentially objective assessment methods might be more suitable for making comparisons across populations (25).

Studies have consistently shown that advancing age is a risk factor for asthenopia (4, 21). Univariate factor analysis showed increasing age, chief doctors and presbyopia were risk factors associated with asthenopia in this study. However, presbyopia remained to be a risk factor, increasing age and chief doctors were not related after multivariate analysis to adjust the potential danger of co-variation between variables. It may be due to presbyopia being the key factor to asthenopia, increasing age and chief doctors may lead to presbyopia. There is no doubt that doctors will achieve higher physician levels with the increasing age. Meanwhile, eye accommodation declines with age, and individuals over 40–45 years old are susceptible to asthenopia after prolonged near-distance work (4, 17, 18).

In this study involving Chinese ophthalmologists, multivariate analysis revealed that ophthalmologists working at tertiary hospitals had a 12% lower risk (OR = 0.88, *p* = 0.027) of experiencing asthenopia compared to those in non-tertiary hospitals. Despite ophthalmologists at tertiary hospitals spending more time on near vision tasks and less time on sleep (both of which were identified as risk factors for asthenopia in this study), a notably smaller proportion of them suffered from asthenopia compared to their counterparts at non-tertiary hospitals (asthenopia prevalence: 39.3% vs. 42.1%, *p* = 0.043). The unexpected outcome prompts an exploration of potential reasons. (1) Ophthalmologists at tertiary hospitals were younger (34.9 ± 8.6 years vs. 35.6 ± 8.5 years, *p* = 0.008) than those at non-tertiary hospitals, and lower percentage of presbyopia (17.2% vs. 19.1%, *p* = 0.078) although two groups showed no statistical difference. Ophthalmologists can identify and manage age-related eye issues promptly, presbyopia associated with increasing age remains a risk factor for asthenopia among them. (2) Researches (6, 13) indicate that good mental well-being guards against asthenopia. Francesca’s study (26) also demonstrated a significant association between asthenopia and perceived anxiety (OR = 7.40, *p* < 0.001), as well as psychosocial factors (OR = 1.03, *p* = 0.026). We hypothesize that ophthalmologists at tertiary hospitals possess better mental states and greater stress resilience. Lower percentages of experiencing anxiety (46.1% vs. 50.8%, *p* = 0.001) or depression (30.7% vs. 35.0%, *p* = 0.001) due to asthenopia were noted among those working at tertiary hospitals in this study. Wang’s investigation (27) established that the mental health of psychiatric medical personnel at tertiary hospitals surpassed that of non-tertiary hospitals, and they adopted more positive coping strategies. Naturally, this implies that beyond age, near vision activity duration, and sleep, other factors influence asthenopia. Future researches should encompass more potential factors to thoroughly explore the influencers of asthenopia in ophthalmologists.

Asthenopia, influenced by both mental and physical factors, reveals a significant insight in our study, particularly regarding the impact of sleep quality on disease. Our research indicates a dose-related relationship, where moderate sleep quality is associated with a 53% lower risk (OR = 0.47, *p* < 0.001), and good sleep quality corresponds to a 76% lower risk (OR = 0.24, *p* < 0.001) of asthenopia compared to poor sleep quality. This protective effect of enhanced sleep quality is consistent with findings in shift-working nurses (2) and college students (28). Previous studies (29, 30) suggest that although poor sleep quality may not directly cause asthenopia, it can exacerbate the condition by influencing mood and worsening depressive symptoms. Longitudinal investigations also propose that sleep disturbances arising from subpar sleep quality may contribute to depression risk (31). Additionally, symptoms associated with asthenopia, such as dry eyes and headaches, can negatively impact sleep quality (32), exacerbating asthenopia.

Nevertheless, our study has several limitations. We depended on self-reported perceptions of each symptom to assess asthenopia rather than employing objective measurements. Integrating both subjective and objective assessments could have bolstered the study’s effectiveness. In capturing information about potential risk factors, we utilized single-item self-report questions to evaluate the duration of near vision tasks and sleep quality, potentially overlooking important details such as subspecialty, refractive defect, doctor-patient dynamics, and strategies for alleviating asthenopia. Other constraints include the cross-sectional design, preventing the drawing of causal inferences, and potential inaccuracies due to self-reporting. However, our investigation benefited from a substantial sample size, aiming to minimize bias, and relied on the reliable and validated ASQ-11 survey tool. Additionally, this study assessed participants’ subjective symptoms over the preceding 2 weeks to reduce recall bias.

In conclusion, preserving ocular health stands as a crucial occupational priority. This study revealed a 40.7% prevalence of asthenopia among Chinese ophthalmologists, highlighting working at tertiary hospitals as a protective factor and presbyopia as a risk factor. Preventive measures for asthenopia involve improving sleep quality, limiting daily near vision tasks to less than 7 h daily, and ensuring more than 7 h of sleep per day. Subsequent research is needed to delve into the role of tertiary hospitals as a protective factor.

## Data availability statement

The raw data supporting the conclusions of this article will be made available by the authors with the permission of the head of the department, upon reasonable request.

## Ethics statement

The studies involving humans were approved by the Institutional Ethics Committee of the Eye Hospital of Wenzhou Medical University. The studies were conducted in accordance with the local legislation and institutional requirements. The participants provided their written informed consent to participate in this study.

## Author contributions

NL: Funding acquisition, Investigation, Methodology, Writing – original draft. YZ: Investigation, Project administration, Writing – original draft. XW: Investigation, Project administration, Writing – original draft. MY: Investigation, Project administration, Writing – original draft. FL: Funding acquisition, Methodology, Supervision, Writing – review & editing. RD: Funding acquisition, Methodology, Supervision, Writing – review & editing.
